# Comparison of Gross Pathology between Classical and Recombinant Lumpy Skin Disease Viruses

**DOI:** 10.3390/v15091883

**Published:** 2023-09-05

**Authors:** Irina Shumilova, Alexander Sprygin, Ali Mazloum, Valeriy Pronin, Olga Byadovskaya, Shawn Babiuk, Irina Donnik, Ilya Chvala

**Affiliations:** 1 Federal Center for Animal Health, 600901 Vladimir, Russia; shumilova@arriah.ru (I.S.); ali.mazloum6@gmail.com (A.M.); pronin@arriah.ru (V.P.); bjadovskaya@arriah.ru (O.B.); chvala@arriah.ru (I.C.); 2 Canadian Food Inspection Agency, Ottawa, ON K1A 0Y9, Canada; shawn.babiuk@canada.ca; 3 Federal State Budgetary Educational, Institution of Higher Education, Ural State Agrarian University (FSBEI HE Ural SAU), 620075 Yekaterinburg, Russia

**Keywords:** lumpy skin disease virus, lumpy skin diseases, recombinant virus, necropsy, experimental bioassays, pathology

## Abstract

The pathology caused by three different isolates of lumpy skin disease virus, classical field cluster 1.2 strain Dagestan/2015, recombinant vaccine-like cluster 2.1 strain Saratov/2017, and cluster 2.2 strain Udmurtiya/2019, in cattle was compared from experimental infections. The infection of cattle was performed using intravenous administration of 2 mL of 10^5^ TCID50/mL of each specific LSDV. Both classical and recombinant forms of LSDV cause pathological changes in the skin and lymph nodes, as well as the trachea and lungs. Due to circulatory disorders in the affected organs, multiple areas of tissue necrosis were observed, which, with the resurgence of secondary microflora, led to the development of purulent inflammation. Observed pathological changes caused by the recombinant vaccine-like strain Udmurtiya/2019 were characterized by a more pronounced manifestation of the pathoanatomical picture compared to the classical field strains Dagestan/2015 and Saratov/2017. Interestingly, Dagestan/2015 and Udmurtiya/2019 caused damage to the lymph nodes, characterized by serous inflammation and focal purulent lymphadenitis caused by purulent microflora. “Saratov/2017” did not cause pathology in the lymph nodes. All LSDVs were virulent and caused pathology, which was not distinguishable between viruses. This data set will serve as the experimentally validated basis for the comparative examination of novel LSDV strains in gross pathology.

## 1. Introduction

Lumpy skin disease virus (LSDV), belonging to the *Poxviridae* family of the genus *Capripoxvirus*, is the causative agent of lumpy skin disease (LSD). LSD is an emerging transboundary disease that continues to spread, expanding its range in Southeast Asia and posing a serious threat to cattle worldwide [1]. LSDV primarily affects cattle, although water buffaloes, giraffes, and antelopes are also susceptible [2,3]. LSDV outbreaks cause high economic losses due to trade restrictions as well as direct production losses from reduced milk production, emaciated animals, and damage to hides. Hence, an LSDV infection is to be reported to WOAH.

Clinical signs of LSD are characterized by fever and the formation of skin lesions that can extend deep into the underlying tissues, including muscle [4]. The clinical disease spectrum caused by LSDV is variable in both experimentally infected cattle and in outbreaks in the field. Skin lesions contain a high level of virus, serving as a source of contamination for environmental materials. In infected animals, the virus disseminates throughout the body with tropism towards epithelial cells, followed by shedding with oral, nasal, and ocular secretions, as well as semen [5].

The gross pathology caused by LSD is characterized by skin nodules and lesions on the epithelium of the respiratory and gastrointestinal tracts [6]. A study by Sanz-Bernardo et al. 2020 specifically focused on the progression of nodule development, showing severe multifocal dermatitis with necrotizing fibrinoid vasculitis [7]. Unfortunately, studies on gross pathology following LSDV infection are limited to viruses belonging to cluster 1.2 only. The emergence and spread of virulent recombinant LSDV into Russia and China has occurred following the use of a contaminated Kenyan Neethling vaccine in Kazakhstan [8,9]. Recombinant LSDV have some differences compared with classical viruses, including increased growth in cell culture as well as the ability to transmit by contact [10], and it is not known if these recombinant viruses display differences with respect to gross pathology.

The goal of this study was to compare the gross pathology observed during experimental infections with the classic strain Dagestan/2015 and the recombinant vaccine-like strains Saratov/2017 and Udmurtiya/2019 to determine if there are any differences between recombinant and classical LSDVs with respect to gross pathology.

## 2. Materials and Methods

### 2.1. Viruses

In this study, three LSDVs were used: 1—classical field LSDV Dagestan/2015 (MH893760) belonging to cluster 1.2 [11]; 2—recombinant LSDV Udmurtiya/2019 (MT134042) belonging to cluster 2.2 whose genomic backbone is composed of KSGPO-240 vaccine strain as the major component and Neethling LW1959 as the minor component [12]; and 3—recombinant LSDV Saratov/2017 (MH646674) belonging to cluster 2.1 whose genome is composed of Neethling strain genome as a major parent and KSGPO-240 as a minor one [11].

The isolates were subjected to two serial passages in goat testis cells prior to the characterization of the virus genome, as stated in previously published work [11,12]. Virus titration prior to the infection of animals was determined in 96-microwell plates using a 10-fold dilution. The plates were incubated at 37 °C with 5% CO_2_ for 72 h and inspected daily for the presence of a cytopathic effect (CPE). Cell or negative control wells had to demonstrate the absence of CPE, while characteristic CPE in the form of lumps on the cell layer had to be present in wells for the virus or positive control. The virus titer was calculated according to the Spearman–Karber method, as reported previously [11,12]. The results are expressed in logarithms as 50% tissue culture infective dose (log TCID50). 

### 2.2. Experimental Design

LSDV experimental infections were performed using intravenous administration of 2 mL of 10^5^ TCID50/mL of each specific LSDV. Briefly, the experiments were conducted with 6- to 8-month-old bulls in Animal Biosafety Level 3 housing with a 12-h light-dark cycle, a relative humidity of 30% to 70%, and a temperature of 23 °C to 26 °C. All animals were monitored twice daily by the veterinary staff. Postmortem data for Saratov/2017 were collected at day 61 post-infection (p.i.) [13], for Udmurtiya at day 41 p.i. [10], and for Dagestan at day 41 p.i. [4].

## 3. Results

### 3.1. Clinical Disease in Cattle

The clinical signs in experimental animals were described in more detail in previous published manuscripts [4,10,13], with the main clinical signs for each isolate described below.

#### 3.1.1. Dagestan/2015 [4]

Upon examination and necropsy, cattle with generalized disease had skin lesions, noticeably enlarged lymph nodes, and lesions in the lungs, trachea, and testicles, whereas subclinically ill animals exhibited only enlarged lymph nodes and fever.

Following infection, eight animals exhibited increased body temperatures of 40 °C–41 °C between days 6 and 8 p.i., nasal and saliva discharge, and skin lesions on mucosal surfaces of the mouth and nose. Hyperemia was also observed on the conjunctiva. The lymphatic nodes were bulging and sensitive to touch. The animals had pronounced oedema extending down to the dewlap and the distal ends of the extremities. The fur-free body sites (scrotum) and the groin area were covered with skin lesions. Four animals (Nos. 2, 6, 8, and 11) showed subclinical disease characterized by fever and exhibited gross pathology only in the lymph nodes. Two contact control animals, housed with inoculated animals from the beginning of the experiment, remained disease-free throughout the experiment. No infected cattle died from LSDV by day 21 p.i.

#### 3.1.2. Saratov/2017 [13]

There was noticeable edema in the inoculated bulls (IN group) in their subscapular axillary and popliteal lymph nodes, except in bull IN-6—this animal exhibited edema in its dewlap area and joints, accompanied by lesions on its muzzle, and general weakness. In the contact group (C1 group), edema appeared later, between days 28–30 p.i., in the areas under the jaw, corresponding with the detectable viremia and elevated body temperatures in all the bulls in this group (up to 40.6 °C in C1–5). In the C2 group, elevated temperatures were recorded in all animals within a week following their inclusion in the trial (day 33 p.i.) and found to be associated with slightly enlarged lymph nodes and edema in the jaw (for C2–3), and the C2–1 bull displayed a mild edema in its dewlap.

There was a fulminating disease presentation in infected bull Nos. 4, 6, and 10, with symptoms of multiple lumps coalescing together over the entire body (up to 3 cm in diameter), erosions in the foci up to 2 cm in diameter, and lesions in the scapula and sides as large as 1.5 cm in diameter. Erosions were also observed on their muzzles and associated epithelia. Bull No. 8 displayed similar symptoms, but their appearance was delayed. By day 21 p.i., the size of the lesions had enlarged to 2.5 cm in diameter in inoculated bull Nos. 4, 6, and 10, and their nasal epithelia were hyperemic. Also, there were isolated foci of necrotic lesions in the scrota of these animals.

For the C1 bulls, by day 28 p.i., three of them exhibited small erosions in their nasal epithelia and in their muzzles, during which period their viremia was significant with high body temperatures (40.6 °C in C1–5). Importantly, no erosions were evident towards the rear half of their bodies (scrotum or inner sides of their legs), as opposed to small skin lesions present on their necks. Three out of five C1 animals (C1–5, C1–7, and C1–9) developed characteristic signs of viral infection by day 35, with the symptoms, including enlarged lymph nodes (predominantly prescapular, paratracheal, and head lymph glands) and multiple lumps over their bodies, from their heads to their tails, 0.5 to 4 cm in diameter. However, there was no pronounced pathology in the popliteal or groin lymph glands as compared to the inoculated animals, and there were no erosions in their scrotums.

There were variable clinical symptoms in the C2 animals: C2–1 displayed a mild edema in its dewlap, whereas C2–5 developed a few foci of erosion in its scrotum. At post-introduction day 21 (i.e., day 54 of the experiment), the C2–3 bull displayed a few small lumps in the scapular region. By post-introduction day 26, these lumps had increased in number and size, between 2.0 and 2.5 cm in diameter.

#### 3.1.3. Udmurtiya 2019 [10]

The first clinical manifestations of LSD in the form of small skin nodules on the neck and shoulder area, as well as roseola on the scrotum, were observed starting at days 8 and 9 p.i. in animals No. 3, No. 5, and No. 7 belonging to the IN group. This was simultaneous with the increase in body temperature. At day 10 p.i., bull No. 5 was in a state of apathy, displaying a refusal to move and multiple nodules throughout its body. Animals No. 3, No. 5, and No. 7 displayed an increase in superficial lymph nodes, weakness, and heavy breathing. At day 11 p.i., the skin lesions enlarged from 0.3 × 0.3 cm to 4.0 × 4.5 cm in size and covered the entire body. Additionally, at day 11 p.i., the first clinical signs of LSD were detected in the control (C) group with several small nodules, about 0.2–0.3 cm in diameter, forming on the left shoulder blade and left side of the neck of bull No. 8. Five days later (16 dpi), the number and size of nodular lesions increased, reaching a diameter of 0.5–0.7 cm. Additional small nodular lesions were detected on the clavicle area of the same animal (bull No. 8) on day 16 dpi. During this time, inflamed nodular lesions were observed in infected animals (No. 3, No. 5, and No. 7) in addition to erosive skin lesions of the nasolabial folds. Clinical manifestations of LSD were observed in four of the five uninfected (C) animals on day 26 of the experiment. Bulls No. 2, No. 4, and No. 6 displayed severe symptoms, including nodular lesions on their backs, heads, fore and hind limbs, scrotum, erosive lesions on the nasolabial mirror, enlarged lymph nodes, and subcutaneous tissue edema in the submandibular region. In contrast, bull No. 8 displayed a few small, nodular skin lesions on the left shoulder blade and left side of its neck for 10 days, followed by no additional external manifestations of LSD. The infected animals (No. 3, No. 5, and No. 7) continuously displayed clinical signs of LSD as previously described until the end of the experiment on day 42. Additionally, bull No. 7 developed painful edema on the right hind leg, severe weakness, decreased appetite, and exhaustion. Uninfected animals No. 2, No. 4, and No. 6 developed classical LSD symptoms during the progression of the experiment. On day 33, bull No. 2 developed conjunctivitis. Severe disease symptoms were observed in bull No. 4, which included extensive edema on the left half of the animal’s head. The animals No. 1 and No. 9, which were intravenously infected with LSDV Udmurtiya/2019 at the start of the experiment, as well as one of the control animals, No. 10, remained healthy and without visible signs of clinical LSD for the 41-day duration of the experiment.

### 3.2. Post-Mortem Findings

#### 3.2.1. Skin and Muscle

**Figure 1:**
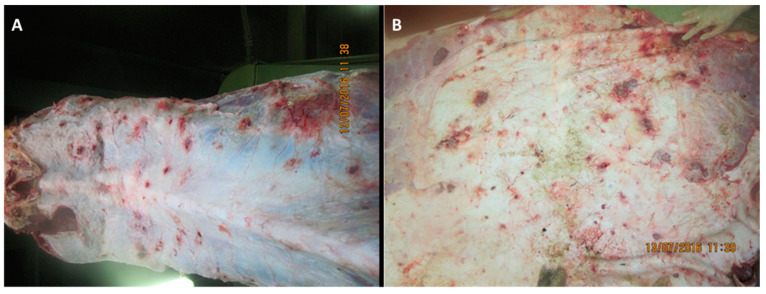
Pathological changes in skin, subcutaneous, and muscle tissues in cattle infected with LSDV Dagestan/2015. (**A**) Multiple focal necrotic dermatomyositis. (**B**) Focal and fine-spotted hemorrhages in subcutaneous tissue. Subcutaneous tissue is moist and shiny, with multiple jagged-edged red foci of various shapes and sizes on the periphery; local, small hemorrhages. Areas with affected outer layers of muscle were observed as red-colored muscle lesions of irregular sizes and shapes.

**Figure 2:**
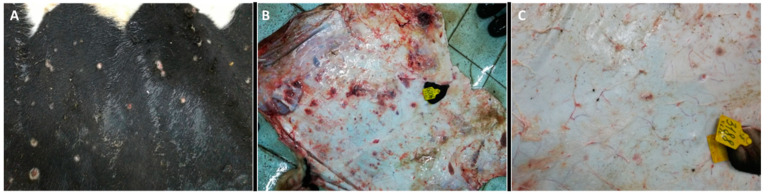
Pathological changes in skin, subcutaneous, and muscle tissue in cattle caused by LSDV Udmurtiya/2019. (**A**) Multiple nodular dermatitis on the skin surface; multiple swollen, dense, rounded-shaped foci of various sizes. In some cases, these areas had raised edges and a concavity in the central part, which is characterized by a rough surface and grayish-red color. (**B**) Multiple focal purulent-necrotic dermatitis (inner surface of the skin). (**C**) Multiple spot and fine-spotted hemorrhages in the subcutaneous tissue; the subcutaneous tissue is wet and shiny, with multiple small-spotted hemorrhages and focal lesions of various shapes and sizes, with uneven edges, purplish-red in color, sometimes with signs of purulent melting.

**Figure 3:**
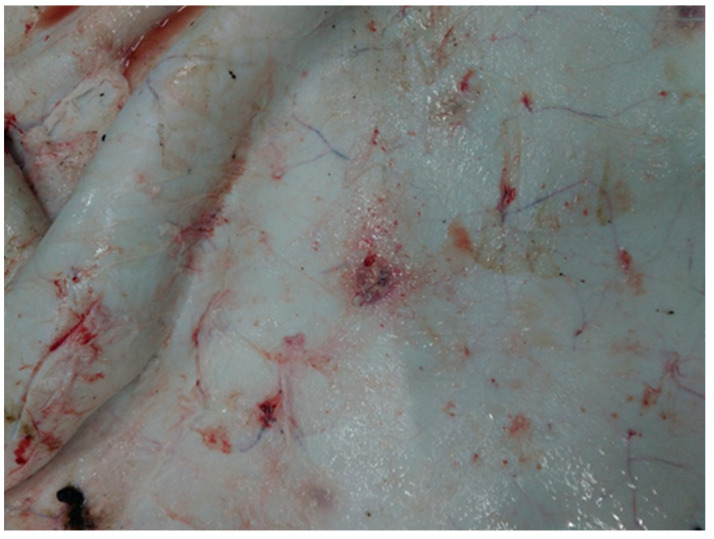
Pathological changes in skin, subcutaneous, and muscle tissues in cattle caused by LSDV Saratov/2017. Focal necrotic dermatitis. Multiple spot hemorrhages in the subcutaneous tissue. Subcutaneous tissue is moist and shiny, has focal lesions of red color, is rounded or irregular in shape, has uneven edges, and has a structureless central part of red-cyanotic color.

Analysis of pathoanatomic changes in the skin, subcutaneous tissue, and musculature of cattle infected with various isolates (Dagestan/2015, Udmurtiya/2019, and Saratov/2017) of LSDV shows that, in all cases, there are pronounced focal changes in the skin and subcutaneous tissue (Figure 1, Figure 2 and Figure 3). In addition, there are abundant hemorrhages from the subcutaneous tissue. The necrotic changes and hemorrhages indicate disorders of the circulatory system, such as the occurrence of thrombosis and increased permeability of blood vessels. Purulent necrosis of a number of necrotic areas in the case of infection with the Udmurtiya/2019 isolate is likely due to an infection with pyogenic microorganisms. It should be noted that when infected with Dagestan isolate, lesions penetrate into the muscle tissue, leading to focal muscle lesions.

#### 3.2.2. Nasal Area

**Figure 4:**
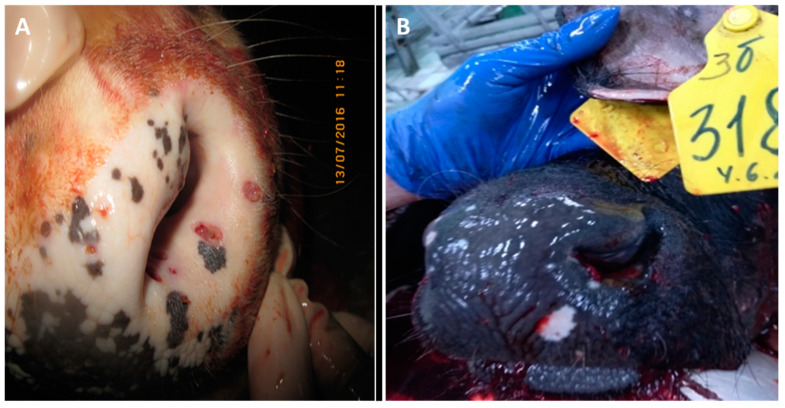
Multiple erosive and ulcerative dermatitis of the nasal mirror skin. (**A**)—Bull infected with Dagestan/2015. The skin of the nasal mirror is moist and shiny, and around it there are multiple defects of a rounded or oval shape with smooth edges; (**B**)—Bull infected with Udmurtiya/2019. Erosive and ulcerative dermatitis of the nasal mirror. On the dorsal surface of the nasal mirror as well as on the inner surface of the skin of the nasal passages, focal lesions of a rounded or oval shape with uneven edges and deepening in the center were found.

The presented photo files, which show pathoanatomic changes in the skin of the nasal mirror of cattle infected with infectious nodular dermatitis (isolates Dagestan/2015 and Udmurtiya/2019) (Figure 4), confirm the violation of tissue integrity not only in the superficial, but also in the deep layers of the skin.

#### 3.2.3. Tongue

**Figure 5:**
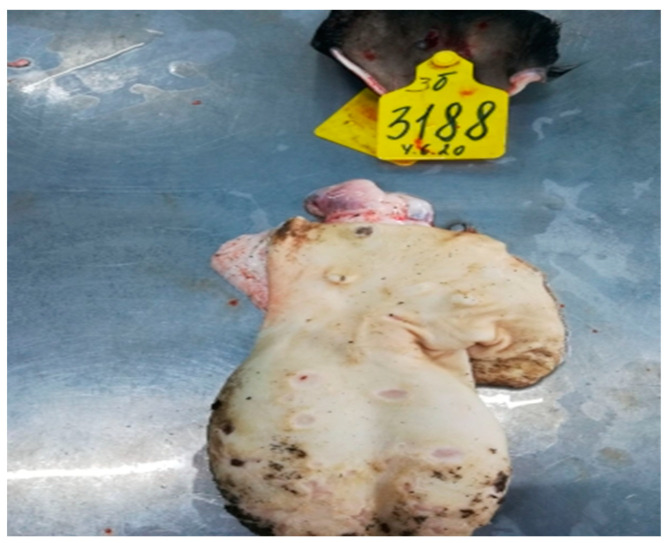
Erosive-ulcerative glossitis—the tongue is enlarged in size and edematous. The mucous membrane is swollen, and in the area of the tip of the tongue, it has focal lesions of irregular shape, merging with each other and penetrating deep into the tissue. On the mucous membrane in the area of the body and the root of the tongue, there are multiple lesions of a rounded shape with towering edges and a sinking center, the surface of which is uneven and grayish-pink in color.

Pathoanatomic changes in the tongue of cattle were observed only when the Udmurtiya/2019 strain was used for infection. Pathoanatomic changes were characterized by lesions of both superficial and deep structural parts (Figure 5).

#### 3.2.4. Lungs

**Figure 6:**
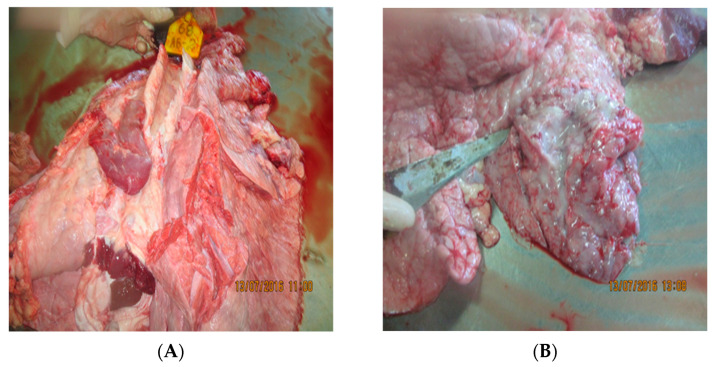
Croup pneumonia complicated by an abscess. Dagestan/2015. (**A**) The lungs are enlarged, bumpy from the surface, and areas of dense consistency are revealed; (**B**) On the incision—moist, the interstitial is expanded, the affected areas are dark red, and, in places, there are cavities filled with purulent contents.

**Figure 7:**
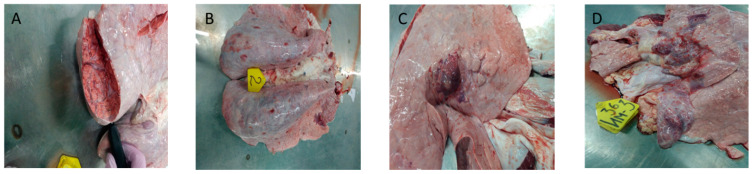
Croup pneumonia by Saratov/2017. (**A**,**B**) Correspond to animals infected intravenously, while (**C**,**D**) are in-contact control animals. (**A**)—Croup pneumonia in animals infected intravenously; (**B**)—The lungs are enlarged, lumpy, and the lung tissue has collapsed in places along the edges; on the incision—grayish-red, the interstitial is expanded; (**C**)—Croup pneumonia by Saratov/2017 with foci of necrosis (in contact animal); (**D**)—Croup pneumonia with foci of necrosis. Some lobes of the lung are enlarged, dark red in color, and dense in consistency. In some places, there are foci of reddish-bluish color protruding above the surface of the lungs and containing structureless grayish-yellowish inclusions (in contact animals).

**Figure 8:**
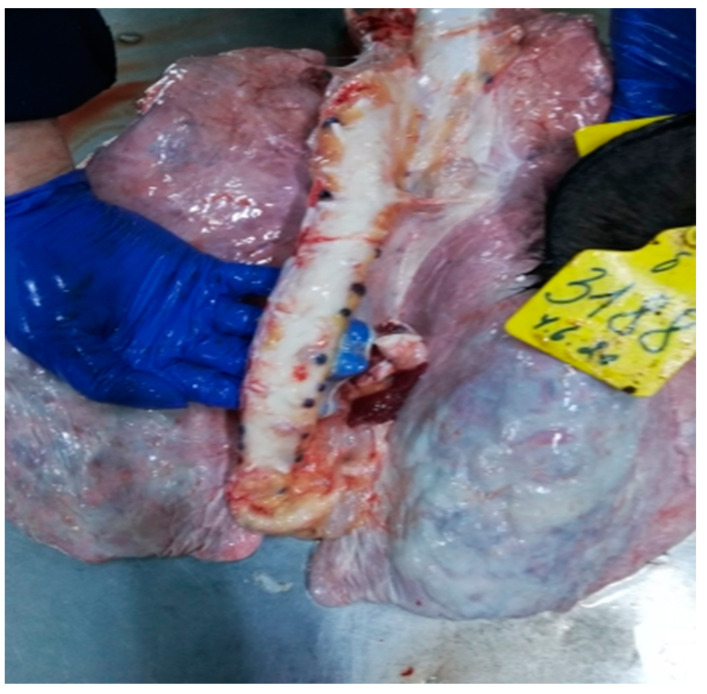
Croup pneumonia. Udmurtiya/2019. The lungs are enlarged in size, bumpy, dense in consistency, and red-bluish in color. The interstitium is expanded and gray-white in color.

In the lungs, when cattle are infected with the Dagestan/2015 isolate, foci resembling abscesses are detected. It should be clarified that a similar outcome with croup pleuropneumonia is possible—purulent melting of the necrosis focus under the action of pyogenic microorganisms (Figure 6). Foci of necrosis are presumably also found in animals infected with the Saratov/2017 isolate (Figure 7). It is impossible to completely exclude the presence of purulent melting of a number of necrotic foci in the case of the Saratov/2017 isolate because it is difficult, and sometimes impossible, to assess the nature of the affected areas on the section from the surface. The absence of any outcomes of fibrinous inflammation in the lungs of cattle in the case of the Udmurtiya/2019 isolate cannot indicate that the process has just begun because there are no more detailed photographs (it is important to know how the altered areas look on the section) (Figure 8).

#### 3.2.5. Trachea

**Figure 9:**
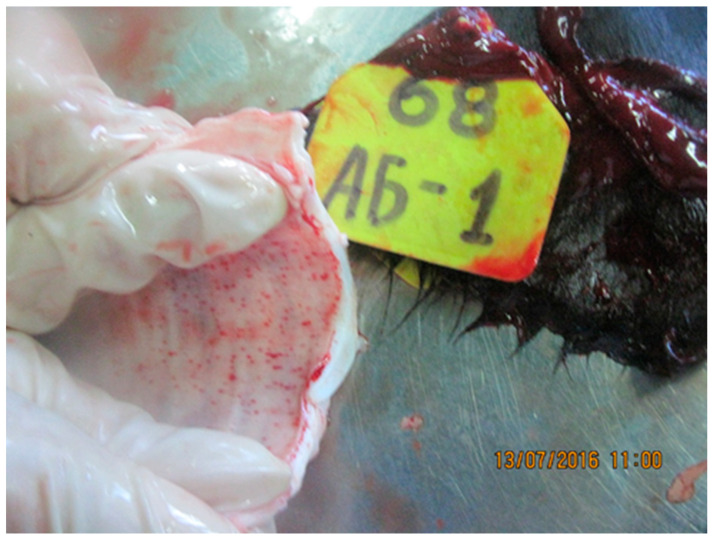
Acute serous tracheitis with multiple spots and small-spotted hemorrhages. Dagestan/2015. The mucous membrane of the trachea is swollen, grayish-pink in color, and, in places, has intense red staining with multiple small hemorrhages.

**Figure 10:**
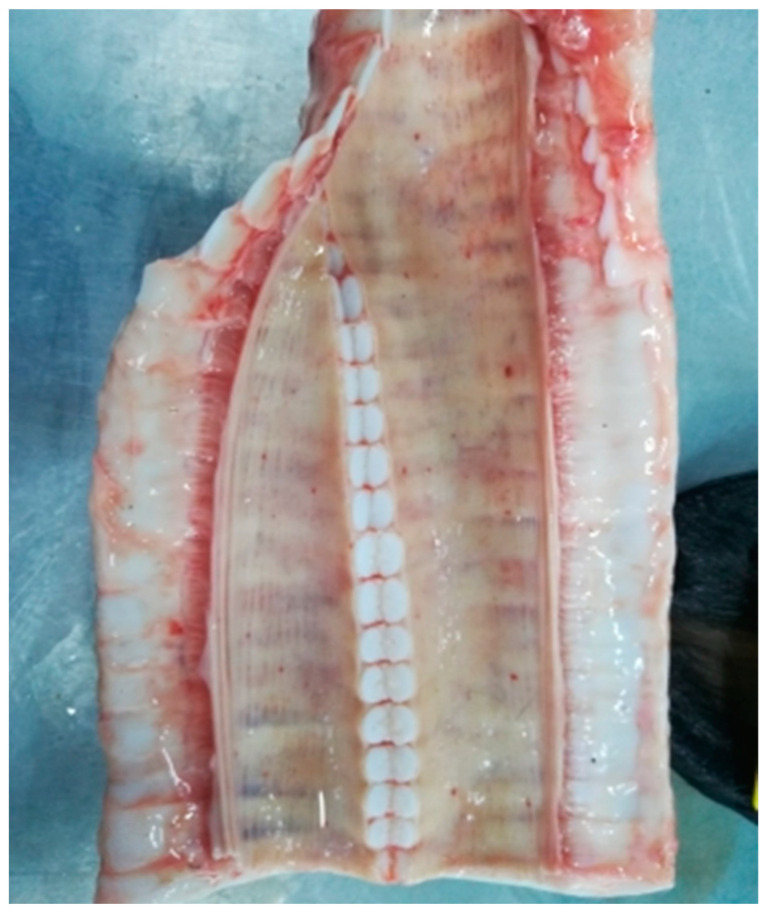
Acute catarrhal tracheitis with single spot and small-spotted hemorrhages. Udmurtiya/2019. The mucous membrane of the trachea is grayish-red in places with a bluish tinge; moist, shiny, straw-colored mucus is noted, as well as spot and fine-spotted hemorrhages. On the mucous membrane in the upper third of the trachea, a large oval-shaped, gray-red focus is visualized, under which the submucosal base is impregnated with a reddish liquid.

The pattern of infectious nodular dermatitis in the case of infection of cattle with isolates Dagestan/2015 and Udmurtiya/2019 is characterized by a tracheal lesion (Figure 9 and Figure 10, respectively). Presumably, as far as it is possible to estimate from the photo (Figure 9), when infected with the Dagestan isolate, the process is quite acute, which is manifested by acute serous inflammation and multiple hemorrhages. When infected with the Udmurtiya/2019 isolate, there are isolated hemorrhages, and the exudate is characterized by the addition of a mucous component. There is no information confirming the pathology of this organ in the case of infection with the Saratov/2017 isolate.

#### 3.2.6. Lymph Nodes

**Figure 11:**
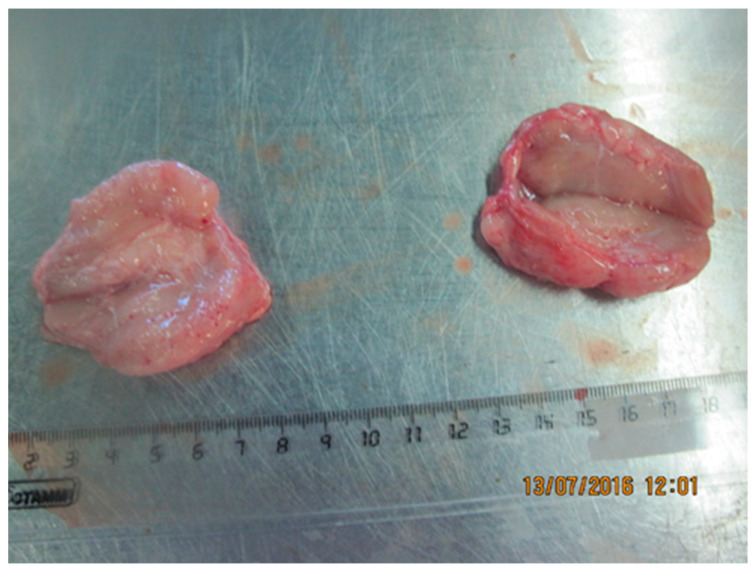
Serous lymphadenitis of the pharyngeal lymph nodes with spot hemorrhages. Dagestan/2015. Lymph nodes are enlarged, juicy, gray-white in color, and have spot hemorrhages.

**Figure 12:**
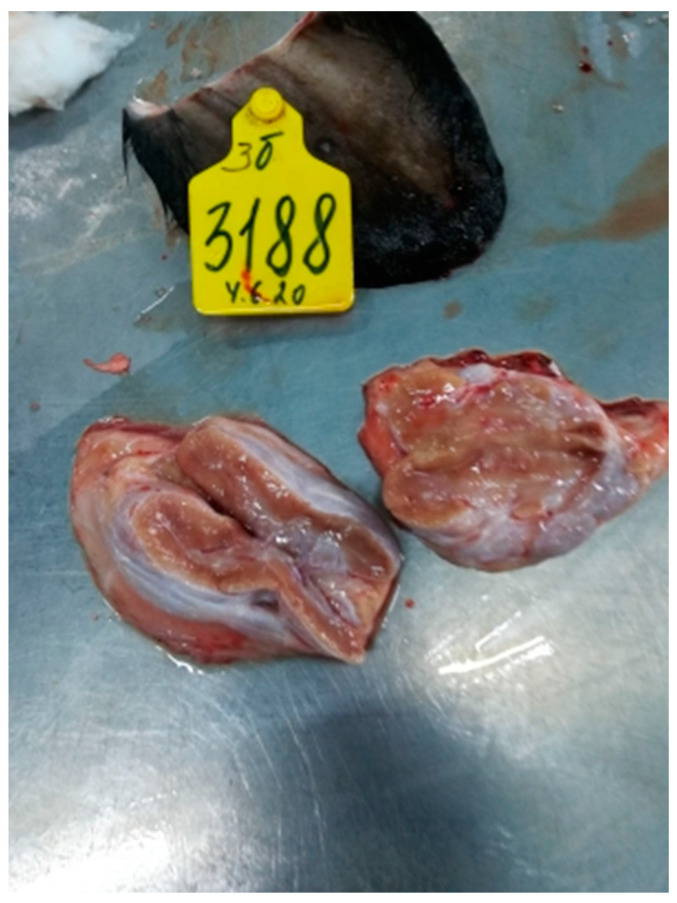
Serous lymphadenitis. Udmurtiya/2019. The lymph node is enlarged in size, moist, shiny, and reddish-brown in color on the incision.

**Figure 13:**
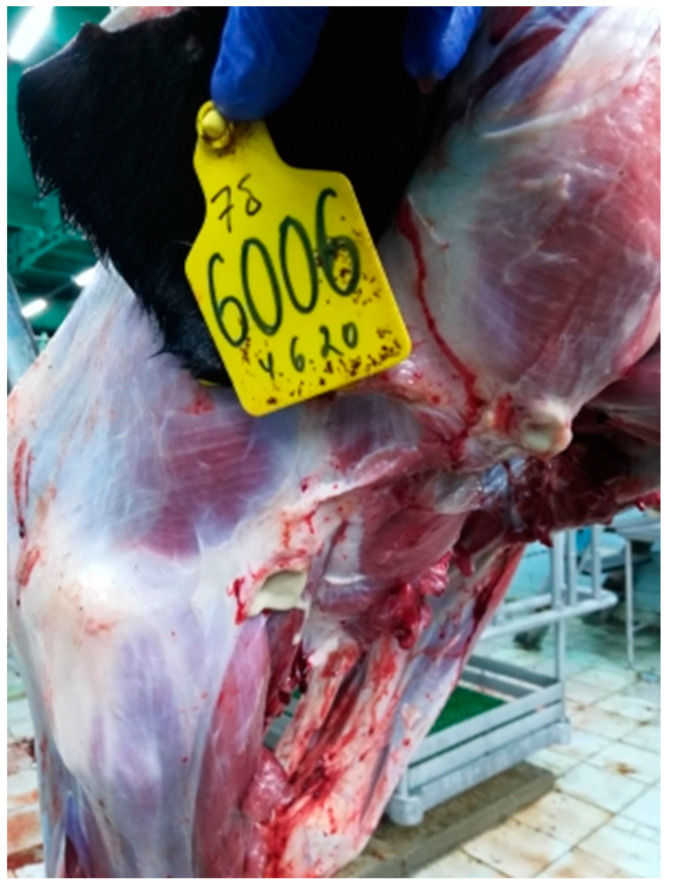
Focal purulent lymphadenitis with Udmurtiya/2019. The lymph node is enlarged, with round and oval foci filled with purulent contents.

The isolates of Dagestan/2015 and Udmurtiya/2019 caused damage to the lymph nodes, and, in the case of the addition of purulent microflora, purulent exudate is formed (Figure 11, Figure 12 and Figure 13). When cattle were infected with the Saratov/2017 isolate, no damage to the lymph nodes was observed.

Pathological changes observed in the skin, subcutaneous tissue, and musculature of cattle infected with various strains of LSDV demonstrate that, in all cases, there are pronounced changes in the skin and subcutaneous tissue in the form of focal necrotic dermatitis and spot or fine-spotted hemorrhages in the subcutaneous tissue. Isolates “Dagestan/2015 and ”Udmurtia/2019” caused damage manifested by erosive and ulcerative dermatitis. In the case of infection with Udmurtia/2019, purulent melting of necrosis foci is noted, probably due to a complication by pyogenic microorganisms. When infected with Dagestan/2015, the changes are deeper and are characterized by damage not only to the skin and subcutaneous tissue, but also to muscle tissue, up to focal necrotic myositis. In cases of infection with Udmurtia/2019, changes develop in the area of the tongue and are characterized by both superficial mucosal defects (erosion) and deeper disorders (ulcers).

Along with skin lesions, subcutaneous tissue, muscles, and visible mucous membranes, the pathological process is also noted in the respiratory organs—the trachea and lungs. The lungs presented fibrinous inflammation (croup pneumonia/pleuropneumonia). When infected with Dagestan/2015, foci resembling abscesses were observed. A similar outcome with croup pleuropneumonia is possible through the action of pyogenic microorganisms. Foci of necrosis, presumably, occur in the case of infection with Saratov/2017. When infected with Udmurtia/2019, it is impossible to reliably confirm or deny the presence of necrotic foci. The pathological picture of LSD, in the case of infections of cattle with either Dagestan/2015 or Udmurtia/2019, is characterized by a tracheal lesion. When infected with the strain Dagestan/2015, the process is quite acute, characterized by acute serous inflammation and multiple hemorrhages; Udmurtia/2019 infection is characterized by single hemorrhages, and exudate is characterized by the addition of a mucous component (catarrhal tracheitis). When infected with Saratov/2017, there were no visible changes in the tracheal mucosa, but a violation of the integrity of the esophageal mucosa in the form of superficial (erosion) and deep (ulcers) defects was observed.

Dagestan/2015 and Udmurtia/2019, in addition, are capable of causing damage to the lymph nodes, characterized by serous inflammation and, in the case of the addition of purulent microflora, focal purulent lymphadenitis. When infected with “Saratov/2017”, no damage to the lymph nodes was observed.

## 4. Discussion

Classical LSD symptoms such as anorexia, fever, enlargement of lymph nodes, appearance of skin nodules covering either the entire body or parts of the body, lameness, and swelling of the fetlock and hock joints are commonly detected in field outbreaks and controlled experiments [14,15,16]. Thus, the farmers and outbreak investigators easily learned to identify the typical clinical signs of LSD. According to the published data, the duration of illness and the total length of persistence of skin nodules were almost similar, usually between two and four weeks [17].

However, the appearance of recombinant variants of LSDV raised the question of any clinical signs and pathology that could be differential between classical and recombinant viral infections. Since recombinant vaccines like LSDV strains exhibit features like contact transmission or more aggressive growth [10,13,18], gaining insights into the pathology caused by different lineages can provide clues on LSDV biology. Currently, limited literature is available regarding the variation in clinical signs and pathology caused by classical and recombinant LSDV. Although experiments have been performed with different isolates to assess the clinical symptoms and virus shedding, limited gross pathology findings have been reported for recombinant LSDVs [4,19,20,21]. Nevertheless, hardly any study looked into the comparison of post-mortem findings in LSDV experiments.

In this study, for the first time, we compared the pathology of LSDV following experimental infections of cattle with the classical LSDV Dagestan/2015, LSDV Saratov/2017, and LSDV Udmurtiya/2019 [4,10,13]. Importantly, these viruses belong to three different genetic lineages: classical field cluster 1.2 strain Dagestan/2015, recombinant vaccines like cluster 2.1 Saratov/2017 (major parent—Neethling, minor parent—KSGP), and recombinant vaccines like cluster 2.2 strain Udmurtiya/2019 (major parent—KSGP, minor parent—Neethling) [9,11,12]. The obtained results demonstrate that both classical and recombinant LSDVs cause pathological changes not only in target organs (skin and lymph nodes), but also in the respiratory system.

The findings from the three experiments reveal that the observed gross pathology was similar between classical and recombinant LSDVs, and it was not possible to determine whether an animal was infected with the classic or recombinant LSDV. This means that recombinant strains are as pathogenic as parental strains but carry features absent from their parental counterparts [22,23]. Traditionally, LSD ulcerative lesions can be observed in the oral cavity, tracheal mucosa, and lungs, as described in field manuals (https://www.fao.org/3/i7330e/i7330e.pdf (accessed on 23 June 2023)). Unfortunately, in the available literature, despite the abundance of publications on LSD, the clinical gross pathology caused by recombinant LSDVs has been lacking, significantly limiting the understanding of the pathogenesis of LSDVs [7,19].

In this work, gross pathology of the skin, subcutaneous tissue, and skeletal muscles of cattle infected with Dagestan/2015 and Udmurtiya/2019 shows that all animals had pronounced focal changes in the skin and subcutaneous tissue. LSDV is known to cause characteristic multifocal cutaneous nodules [7]. In addition, Dagestan/2015 and Udmurtiya/2019 induced abundant hemorrhages in the subcutaneous tissue. This may be due to host genetics that play a role in the clinical manifestation of LSD and its variation across the set of experimental animals [24,25]. Interestingly, multiple hemorrhagic and necrotic changes indicate disorders of the circulatory system of infected animals, such as thrombosis, increased permeability of the walls of blood vessels, etc. [7]. Purulent melting of necrotic areas is probably caused by complications of pyogenic microorganisms [26]. It should be noted that in cattle infected with Dagestan/2015, the inflammatory process could reach into underlying muscle tissue due to focal muscle lesions developing [4].

In our study, lesions were also present in the trachea in all infected animals, which is not routinely reported in field cases. At the same time, however, when infected with classical field Dagestan/2015, the inflammatory process of the tracheal mucosa was quite acute, which is manifested by acute serous tracheitis and multiple hemorrhages. However, when infected with the recombinant LSDV Udmurtiya/2019, only single hemorrhages were observed, and the exudate was characterized by the presence of mucous. These different gross pathology findings may indicate either different pathogenesis factors employed by the two strains or an interplay between the virus and host genetics.

Another important observation is fibrinous inflammation in the lungs of cattle. As for LSDV Dagestan/2015, it caused purulent foci resembling abscesses in the lungs, which are formed as a result of the purulent melting of necrosis foci in croup pneumonia caused by pyogenic microorganisms. Of note, foci of necrosis were also found in cattle infected with LSDV Udmurtiya/2019, although abscesses in the lungs were not detected in animals of group No. 2. This also raises the concern that the host-virus interaction in LSD deserves a much deeper investigation to discover which host factors may contribute to a pathogenic or subclinical phenotype [10].

It is important to note the difficulty in specifying the stage of the process due to the fact that the photographs mainly show the lungs from the surface. To make a more accurate pathoanatomical diagnosis, it is necessary to evaluate the organ not only from the surface, but also from the inside. In addition, it is important to see which lobes are affected, which structures are involved in the process, and to assess the consistency of the damaged areas, color, etc. Furthermore, it is also difficult to give an objective assessment of the condition of the pleura. These questions go beyond the scope of this work and deserve further study.

The examination of the lymph nodes shows that LSDV Dagestan/2015 and LSDV Ud-murtiya/2019 caused enlargement and damage to the lymph nodes, with purulent exudate forming where purulent microflora were present. The enlarged nodes are a good sign that the animal is subclinically infected with LSDV [23].

The literature search did not find similar studies, and our reported gross findings on three strains are difficult to interpret and place in the context of available knowledge because the available literature lacks this kind of evidence. Our literature search identified a few studies that generally report on internal lumpy skin lesions. Tuppurainen et al. 2017 discovered ulcerative lesions in the oral cavity and lesions in the trachea, which is in agreement with our data; however, the authors also found gross pathology in the gall bladder—the body site where we did not observe anything [27]. Other organs of the gastrointestinal tract can have lesions caused by lumpy skin disease [19], although this was not observed in this study.

In conclusion, the principal finding is that there is variability in the presentation of gross pathology between individual animals following infection with different LSDVs. These differences between animals were observed in Dagestan/2015, Saratov/2017 and Udmurtiya/2019. However, all these viruses caused similar gross pathology in the primary target organs, although it was not possible to specify the LSDV lineage causing the infection using the observed findings.

## Data Availability

All data reported in this study are available within the manuscript.
